# Navigating the Credibility of Web-Based Information During the COVID-19 Pandemic: Using Mnemonics to Empower the Public to Spot Red Flags in Health Information on the Internet

**DOI:** 10.2196/38269

**Published:** 2022-06-17

**Authors:** Jessica Stokes-Parish

**Affiliations:** 1 Faculty of Health Sciences and Medicine Bond University Robina Australia

**Keywords:** science communication, critical appraisal, social media, health literacy, digital literacy, misinformation, COVID-19, online health, infodemic, infodemiology

## Abstract

Misinformation creates challenges for the general public in differentiating truth from fiction in web-based content. During the COVID-19 pandemic, this issue has been amplified due to high volumes of news and changing information. Evidence on misinformation largely focuses on understanding the psychology of misinformation and debunking strategies but neglects to explore critical thinking education for the general public. This viewpoint outlines the science of misinformation and the current resources available to the public. This paper describes the development and theoretical underpinnings of a mnemonic (Conflict of Interest, References, Author, Buzzwords, Scope of Practice [CRABS]) for identifying misinformation in web-based health content. Leveraging evidence-based educational strategies may be a promising approach for empowering the public with the confidence needed to differentiate truth from fiction in an infodemic.

## Introduction

### Overview

Recognizing misinformation in web-based content is becoming increasingly difficult. The general public struggles with differentiating credible health information from fiction, but we do not know how best to equip them to do so. In a world where information is at our fingertips, differentiating fact from fiction is a priority. This paper explores the science of misinformation and proposes an accessible framework for identifying misinformation in health content on the internet.

### Background

The COVID-19 pandemic triggered an overabundance of information, including false and misleading information that has contributed to confusion, mistrust, and risk-taking behaviors. This kind of information excess is defined as an *infodemic* [1,2]. From daily press conferences to viral videos, health professionals and the general public alike have struggled to keep up with the overload of health information. The inundation of misinformation, disinformation, and contradictory information has obscured access to credible information.

Misinformation in science communications is not a new thing. *Misinformation* is defined as the inadvertent sharing of false or misleading information, whereas *disinformation* is the deliberate sharing of false or misleading information with the intent to harm [3]. Both topics are of great interest to psychologists and researchers. Prolific misinformation researchers Lewandowsky and colleagues [4] suggest that misinformation may arise when the situation is evolving or when the information is piecemeal. This is certainly the case with the pandemic, during which we have seen changes in information that was correct at a certain time, such as the use of masks to prevent SARS-CoV-2 transmission. Other sources of misinformation include rumors, politicians and governments, vested interests, and the media [4,5].

### The Landscape of Misinformation

Misinformation is shared on a variety of platforms—Twitter, Reddit, WhatsApp, and Facebook to name a few [6]. However, misinformation is not limited to social media; it is also present in traditional media platforms, such as articles in magazines, on websites, and on the news. For example, in an analysis of health information on the internet, researchers found that of 1300 websites on safe infant sleep, only 43.5% provided correct information [7]. In another study on conception information, only 1 in 2 websites contained accurate information on conception [8]. The examples go on and on, particularly in the case of COVID-19, with multiple accounts of misinformation regarding COVID-19 treatments [9-11].

Topics of misinformation occur in a wide variety of fields, such as health and climate sciences [3]. Although it is difficult to quantify which topics have the most focus, we can get an indication by looking to research. Most research related to health misinformation focuses on vaccines, communicable (eg, HIV or COVID-19) and noncommunicable diseases (eg, cancer and diabetes), drugs (eg, tobacco), treatments, autism, and eating disorders [3,6,12].

Although misinformation pertains to the inadvertent sharing of false or partially false information, there is a more sinister kind of misinformation—disinformation. *Disinformation* describes sharing false or partially false information with the intent to harm or profit [13] (the term *fake news* is not used in this summary, as it is not supported by literature surrounding false information). Disinformation is a type of warfare strategy that has been linked to creating confusion regarding vaccination and disrupting election campaigns, as well as issues such as climate change [13,14].

### How Does Misinformation Spread?

There are several decades of research dedicated to this issue, so this viewpoint will not attempt to cover the breadth of research on this complex issue. Instead, this paper will briefly outline why misinformation might spread. The author of this paper considers the following two broad categories of reasons: external and internal reasons. Externally, social media platforms amplify misinformation and disinformation due to their reach and the complex algorithms at play [4,5]. Internally, misinformation and disinformation disrupt our cognitive processes, fragmenting our ability to think logically. The little we do know about how and why misinformation spreads is that it is most often spread by individuals who hold positions of influence (eg, social media influencers or politicians) and share messages with personal opinions and strong negative tones [15]. In addition, a person’s relationship with, or their view of, an individual sharing a piece of information influences perceived credibility; that is, if a person likes the individual and knows them well, the person is more likely to believe the information shared and is less likely to do a credibility check [4,15]. Misinformation is amplified by the impact of confirmation bias; people are more prone to misinformation that supports their worldview or ideology [16,17].

### Health and Digital Literacy in an Infodemic

Although technology platforms such as Facebook and Twitter have a role in curbing the proliferation of misinformation and disinformation, digital literacy and health literacy are key factors in slowing the spread of misinformation and disinformation. *Health literacy* can be defined as the “ability of an individual to obtain and translate knowledge and information in order to maintain and improve health in a way that is appropriate to the individual and system contexts” [18]. Coldwell-Neilson [19] defines *digital literacy* as “the ability to identify and use technology confidently, creatively and critically to meet the demands and challenges of living, learning and working in a digital society.” People with lower health literacy seek out health information less often and have a lesser ability to interpret health messages [20]. We also know that those with lower digital literacy are less able to identify reliable news sources or manipulated images [16], and those with less digital and health literacy are more likely to share false information [21].

### What Is the Solution?

As the infodemic is unlikely to disappear anytime soon, we must consider ways to approach information on the internet. We are quick to defer to experts or exclaim “trust the science” as a sort of mantra for ordinary people. This does not engender trust or transparency in science but rather undermines attempts to engage in conversation about science, reinforces harmful hierarchies, and even leads to people falling for misinformation [22]. This mantra ignores the complexities and nuances of trust and engagement with scientific evidence, such as the influence of political persuasion, worldviews, and personal experiences [23]. Instead of restricting autonomy to that of scientists, it is the suggestion of this author that we consider ways to improve digital and health literacy to empower the general public to make informed decisions about the information they read [24].

### What Exists?

Several resources on digital and health literacy exist. A quick keyword search of *health literacy course* and *health literacy training* on Google highlights the variety of resources from universities and not-for-profit organizations. For example, ScienceUpFirst—an initiative borne out of the COVID-19 pandemic—focuses on credible pandemic information [25]. Although they have a page on credible sources, this page focuses on who ScienceUpFirst considers credible as opposed to identifying components of credibility [25]. In a 2020 systematic review, researchers found that very little research focuses on critical thinking; even then, the limited research focused on student populations as opposed to the general public [26]. In addition, many courses on digital literacy, health literacy, or critical appraisal are recommended to health professionals, such as the Centre for Culture, Ethnicity and Health’s courses [27] and Cochrane Training [28]. Research on misinformation extensively explores debunking, fact-checking, and prebunking (ie, preparing a viewer for incoming misinformation) [4,5]. To improve the health literacy of the general public, we should provide accessible appraisal resources, thereby allowing individuals to feel empowered when it comes to health information. In keeping with the constructivist philosophy, the framework presented herein proposes that the general public should become collaborators in critical appraisal.

## Methods and Theoretical Framework

### Overview of Mnemonic Development

Drawing from the constructivist lens (ie, knowledge is subjective and informed by experiences), this paper considered the literature on credibility and critical appraisal and drew from this author’s expertise as an educator to develop a mnemonic [15]. A mnemonic is a specific strategy for enhancing memory with the aim of improving the recall of information [29]. The purpose of the mnemonic in this instance was purely to create a memorable word (and visual) and a mental model for assessing health information on the internet [30,31].

### The Framework

**Figure 1:**
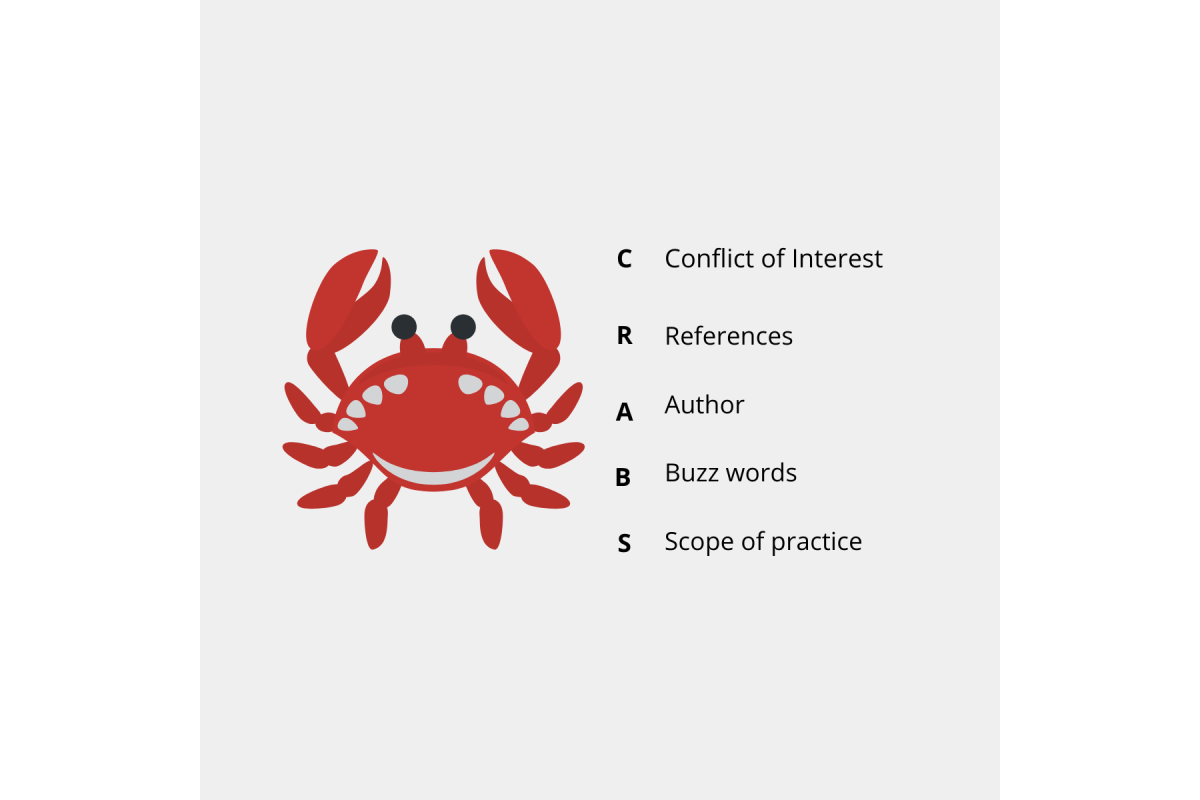
Illustration of the CRABS framework for credibility. CRABS: Conflict of Interest, References, Author, Buzzwords, Scope of Practice.

The mnemonic was developed by using an iterative process. This included an unstructured review of teaching materials for undergraduate and postgraduate health professions education (ie, materials that were used for teaching at the time of writing this paper), the use of library guides, and subsequent crowdsourcing on social media platforms [32-34]. Questions such as “how do you flag questionable content online” and “how would you review content online for accuracy” were used to engage readers. This process resulted in the development of the mnemonic *CRABS* (Conflict of Interest, References, Author, Buzzwords, Scope of Practice; Figure 1).

This paper’s author presented the framework development work at professional development events and published it on several social media platforms. This was presented to registered nurses in Australia for a professional development activity on exploring credible content in the media. The feedback was overwhelmingly positive regarding the mnemonic, with 70% of participants identifying the mnemonic as their key takeaway from the activity. At the time of writing this paper, the framework has had significant reach on this author’s social media platforms (Table 1). In addition to this, the work has been amplified on other social media influencers’ posts, culminating in 68,000 unique views, and translated in other languages [35-37].

In addition to this, the work has been published on various media platforms, such as the Australian Broadcasting Corporation, lifestyle magazines, and high school education resources [38-40]. In health care, the framework has been shared in professional development resources, so that health educators can use the framework for their programs [41,42].

Now, we move on to the framework and underpinning rationale.

**Table 1:** Summary of impact.

Platform	Likes, n	Shares, n	Saves, n	Total number of unique views
Instagram	3783	1280	1691	54,492
Twitter	27	14	N/A^a^	8330

^a^N/A: not applicable.

### C—Conflict of Interest

Conflicts of interest occur when an individual stands to benefit from a certain message or decision, making the information less reliable. Conflicts can be overt or subtle [43]. One example is an individual who owns a nutrition supplement company. This is an overt conflict of interest, as they are likely to prefer their product over others because it benefits them financially. In a more subtle example, a physician may have a family member who owns a company that manufactures wound care products; while there may not be any formal agreement, this relationship may influence the decisions that the physician makes about wound care [44]. In research, conflicts of interest may undermine the validity of results and undercut integrity. There have been many reports of trial sponsors inducing favorable results in research [45]. Conflicts of interest are not limited to finances; they can also include conflicts related to politics, policies, and employment [44]. Conflicts of interest should be considered when judging health information on the internet.

### R—References

References are a useful gauge of content on the internet, as they indicate several things—supporting data, the body of evidence, the quality of evidence, and plagiarism [46]. Supporting data are obvious to scientists; one cannot make a claim without evidence. However, in health information on the internet, particularly on social media, the use of references is less common. Reminding viewers to consider references may assist them in considering the weight to give a claim. Additionally, references can be a good indication of whether the content author has training and an understanding of the body of evidence related to the topic. For instance, authors not citing sentinel work in their blogs can be a red flag for incomplete information. In addition to these checks, references should be checked for recency (science changes fast) and the quality of scientific sources. The issue of predatory publishing is not a small one. Predatory publishers accept manuscripts for a fee without performing any quality checks, thereby allowing poor-quality research or misinformation to proliferate [47]. Finally, plagiarism is not limited to scientific mediums; social media is rife with instances of content thieving and misattribution [48,49].

### A—Author

Anyone can write about anything. The internet provides opportunities for everyone to have a voice and has fewer gatekeepers than traditional media [50]. A person’s expertise and qualifications (or lack thereof) in relation to a topic is important to consider when determining how much weight to give to content. Social media verification, whereby an account is given a blue tick to verify that they are indeed a real person, is not an indicator of credibility in the traditional sense. Credibility literature states that there are 5 things to consider with regard to credibility—accuracy, authority, objectivity, currency, and scope [50]. The *Author* item of the CRABS framework encourages readers to check an individual’s training, qualifications, and credentials. In 5 to 10 minutes, a reader can verify qualifications, explore the level of training that an individual has, and learn about the views of an author’s peers (ie, their views on the author). For instance, if an author claims that they conduct research in a given field, the number of publications on the topic that are under their name should be considered.

### B—Buzzwords

Buzzwords are words or phrases that have become fashionable by being used often, but they sometimes have little meaning. Buzzwords, or overly jargon-filled words, are not always designed to deceive people, but they can be used to mislead people. For example, when food packaging includes buzzwords such as *organic*, consumers are more likely to believe that a product is healthy—the health halo effect [51]. Linguistics research argues that clues are in the language used; emotional language is an indication that information is not credible [24]. News that is inaccurate or fake is more likely to use adverbs and verbs and present information with more certainty. This makes it challenging for credible science information, which frequently hedges certainty and does not overstate claims, to compete against noncredible information [52]. Other work suggests that framing the information in a certain way is a key for identifying misinformation. For example, topics of personal concern (eg, health information), emotive topics (eg, one’s children), and the use of personalization pronouns (eg, *you*) can influence readers [17,53]. Overall, the trigger word *buzzwords* may help an individual to scan for jargon, marketing strategies, and emotional language that might frame their perception.

### S—Scope of Practice

The scope of practice describes the practice of a profession that combines an individual’s qualifications and expertise, the setting of practice, and the needs of clients [54]. In a health care setting, it is difficult to overreach the scope of practice due to highly regulated workforces. However, on social media, the scope is mostly unmonitored (but not necessarily unregulated). Most do not set out to overreach their scope of practice; however, it is a slippery slope. A nurse providing specific nutrition advice for newborns may be inappropriate if the nurse has not undergone additional training, depending on the situational context. In addition, it is easy to overstate expertise or specialty due to the halo of authority portrayed on social media. For example, a junior physician can inadvertently portray themselves as an expert in hormones while not having completed their endocrinology training.

## Framework Application and Implications

The CRABS framework is intended to be applied as an overarching concept at a first glance of web-based content. It is not intended to be a full critical appraisal and may inadvertently exclude key qualities of appraisal that would be otherwise identified. One limitation may be that the framework could inadvertently exclude information that is credible due to the piecemeal nature of social media.

There are opportunities for expansion. For example, to verify the quality of the work, further research should be undertaken to determine content validity. Content validity analyses could include expert consensus methods, such as the Delphi method. Following this, consumer representatives (eg, members of the general public from a wide variety of demographic populations) could be engaged to rate the usability of the framework in assessing web-based health information. The framework should be further assessed for reliability and construct testing to ensure that the framework can indeed be used to identify accurate and false information and that it works for various users. Once the work has been validated, it could be used in critical appraisal guides, misinformation resources, and educational campaigns.

Although anecdotal, feedback has suggested that the content of the framework is representative of the issue and that it is usable among various users; however, more work is required to fully develop the framework. In its present state, the work could be presented as a conceptual model for assessing web-based health information, serving as a trigger for critical appraisal. Despite its origins in the COVID-19 pandemic, the work has scope for application beyond this. Areas rife with misinformation (eg, infant sleeping information, as identified earlier in this viewpoint) could be relevant areas.

## Conclusions

In this era of infodemia, the general public requires accessible tools to navigate health information on the internet. Drawing from misinformation and educational research can provide us with tools to navigate this complex issue and develop resources. Using mnemonics is a practical strategy for encoding memory and developing mental models for critical appraisal. The CRABS model may provide a useful strategy for achieving this. More research is needed to explore the validity and usability of such a model for the general public.

## Abbreviations

CRABS: Conflict of Interest, References, Author, Buzzwords, Scope of Practice
